# Determinants of Outpatient Physician Visits in the General Adult German Population during Later Stages of the COVID-19 Pandemic

**DOI:** 10.3390/healthcare10061025

**Published:** 2022-06-01

**Authors:** André Hajek, Hans-Helmut König

**Affiliations:** Department of Health Economics and Health Services Research, University Medical Center Hamburg-Eppendorf, Hamburg Center for Health Economics, 20246 Hamburg, Germany; h.koenig@uke.de

**Keywords:** health care use, health care utilization, Andersen’s behavioral model, COVID-19, coronavirus, SARS-CoV-2, GP visits, specialist visits, physician visits, loneliness, coronavirus anxiety

## Abstract

The goal of this study was to investigate the determinants of outpatient physician visits in Germany during the later stages of the COVID-19 pandemic. Cross-sectional data were used from the general adult population in Germany with *n* = 3091 individuals (data collection in mid-March 2022). Determinants were selected based on the extended Andersen model. The number of GP visits as well as the number of specialist visits in the past 12 months were used as outcome measures. Negative binomial regressions showed that the number of GP visits was positively associated with a lower educational level, being retired, lower levels of loneliness, the presence of at least one chronic condition, lower self-rated health, being vaccinated against COVID-19, and the presence of depression. Moreover, negative binomial regressions showed that the number of specialist visits was positively associated with being female, a lower age, having children, being married, not being full-time employed, the presence of at least one chronic condition, lower self-rated health, the presence of depression, being vaccinated against COVID-19 and having a lower coronavirus anxiety. In conclusion, while our study showed that need factors are still a main driver of outpatient physician visits, our findings additionally showed that predisposing characteristics, enabling resources and psychosocial factors are also important for the number of outpatient physician visits in Germany during the COVID-19 pandemic. Knowledge about these determinants (e.g., vaccination status, loneliness or coronavirus anxiety) is also important to avoid under- or overuse of the healthcare system.

## 1. Introduction

The COVID-19 pandemic may have ramifications for the use of healthcare services, such as the need for capacity adaptation or a reduction of the risk of SARS-CoV-2 transmission. It is critical to understand what factors are associated with health care use (HCU) during the COVID-19 pandemic to avoid inappropriate use of healthcare services. Outpatient physician services (GP or specialist visits) reflect an integral part of HCU. We particularly assume that individuals avoid using outpatient physician services since they may fear an infection with COVID-19 (e.g., while travelling to the doctor or during the waiting time in the practice) [1].

In sum, while several studies exist examining the determinants of postponed HCU and non-utilization in Germany during the pandemic (e.g., [2,3]), there is a general lack of studies investigating the determinants of outpatient physician visits in the general adult population (particularly during later stages of the COVID-19 pandemic). Therefore, the goal of this study was to investigate the determinants of outpatient physician visits in Germany during the later stages of the COVID-19 pandemic. Outpatient medical care is a very important component of the healthcare system in Germany. Such knowledge is of importance since underuse of healthcare services may result in unmet needs. In turn, such unmet needs may markedly reduce health in the long-term. In contrast, knowledge about a potential overuse may help to reduce the economic burden on the healthcare system. For this reason, it is important to clarify the determinants of outpatient physician visits.

With regard to corona-related restrictions and HCU during the pandemic in Germany, strong restrictions (e.g., school closures, travel ban or nightly curfews) were mostly implemented by the federal government in Germany in the years 2020 and 2021. Thus far, studies are mostly missing an investigation of the question of whether outpatient appointments were cancelled in light of the COVID-19 pandemic in Germany. However, prior research has shown that subjective access to healthcare services was quite good [4].

An overview of the most important features of the German health care system can help the reader to better understand the findings of our current study: health insurance is mandatory in Germany’s healthcare system. The vast majority (roughly 90% of the population) are covered by statutory health insurance (SHI), with the remaining 10% covered by private health insurance (PHI). Civil servants, self-employed individuals (e.g., freelancers), and individuals earning more than a certain income threshold can choose between SHI and PHI. Both insurance systems offer comprehensive coverage for medical expenses. Furthermore, access to General Practitioners (GPs) and specialists is guaranteed (no additional requirements), whereas hospital care is only available with a referral from an outpatient physician or in an emergency. Busse et al. provided additional information about the German healthcare system [5].

## 2. Materials and Methods

### 2.1. Sample

The current survey used data from a nationally representative online survey of people aged 18–74 in Germany (total *n* = 3091 respondents). The survey was conducted in Mid-March 2022. Participants were enrolled by the market research firm bilendi & respondi, which is an ISO 26362 certified online sample provider. Respondents were extracted from an online sample so that their age group, gender, and federal state distribution corresponded to the entire German adult population [6]. Based on the socio-demographic data, a random sample of the online access panel was drawn. About 11,900 individuals were invited to participate in this survey, which corresponds to a response rate of about 26%.

All participants provided written informed consent to participate in the study before the beginning of the survey (by agreeing to the online consent form before the survey started), which is a common procedure in online surveys. The Center for Psychosocial Medicine’s Local Psychological Ethics Committee at the University Medical Center Hamburg-Eppendorf approved this study (LPEK-0412).

### 2.2. Outcomes

The number of outpatient physician visits in the previous twelve months and hospital treatment in the previous twelve months were used to calculate HCU. Both variables were self-administered:

- Number of GP visits in the past twelve months
- Number of specialist visits in the past twelve months

Respondents were instructed to count home visits as well. Picking up a prescription should not be counted as a visit to the doctor.

### 2.3. Independent Variables

Grounded on the extended Andersen model [7,8], independent variables were selected. In general, the Andersen model distinguishes between predisposing characteristics (factors such as sex or age), enabling resources (e.g., labor force participation or health insurance) and need factors (e.g., self-rated health or number of chronic conditions). In the extended Andersen model, psychosocial factors can refer to, among other things, specific fears (e.g., coronavirus anxiety).

With regard to predisposing characteristics, we included these independent variables in our study: age, gender (men; women; diverse), presence of at least one child in own household (no; yes), marital status (married, living together with spouse; married, not living together with spouse; divorced; single; widowed), migration background (no; yes), and educational level (Upper secondary school; Qualification for applied upper secondary school; Polytechnic Secondary School; Intermediate Secondary School; Lower Secondary School; Currently in school training/education; Without school-leaving qualification).

With regard to enabling resources, we included labor force participation (full-time employed; retired; other) and loneliness (quantified using the 6-item De Jong Gierveld loneliness tool). By averaging the items, the loneliness score was calculated (ranging from 1 to 4, with higher values corresponding to higher loneliness levels). Cronbach’s alpha was 0.83 in our study.

With regard to need factors, we included the presence of at least one chronic condition (no; yes), self-rated health (from 1 to 5, with higher values corresponding to better self-rated health), probable depression and probable anxiety. The Patient Health Questionnaire-9 (PHQ-9) was used to measure probable depression consisting of nine items (sum score ranges from 0 to 27, with higher values reflecting more depressive symptoms) [9]. In accordance with prior recommendations [10], a PHQ-9 score of ten or higher was used as a cut-off in our study. Cronbach’s alpha was 0.89 in our current study. The Generalized Anxiety Disorder-7 (GAD-7) [11] was used to measure probable anxiety. It consists of seven items (sum score ranges from 0 to 21 with higher values indicating more anxiety symptoms). A cut-off of ten or higher was used as a cut-off in our study [11]. Cronbach’s alpha was 0.91 in our study. Furthermore, being vaccinated against COVID-19 (no; yes) was used.

With regard to psychosocial factors, coronavirus anxiety was included in our regression model. It was quantified using the coronavirus anxiety scale [12,13,14]. Based on five items, a sum score was calculated, which ranges from 0 to 20 (higher values indicating higher coronavirus anxiety). Cronbach’s alpha was 0.92 in our study.

### 2.4. Statistical Analysis

Sample characteristics are first displayed. In a second step, multiple negative binomial regressions were conducted to explore the determinants of the number of (1) GP visits and (2) specialist visits (taking into consideration the distribution of the outcomes [15]).

The significance level was set at *p* < 0.05. Stata 16.1 (Stata Corp., College Station, TX, USA) was used for statistical analyses.

## 3. Results

### 3.1. Sample Characteristics

Sample characteristics are shown in Table 1. Mean age in our sample was 46.5 years (SD: 15.3 years), ranging from 18 to 74 years. In total, about 49.5% were female. In sum, 39.9% of the individuals visited an upper secondary school and 12.0% of the individuals had a migration background. Average GP visits in the past 12 months equaled 2.6 visits (SD: 3.6) and average specialist visits in the past 12 months equaled 2.1 visits (SD: 3.6). Further details are shown in Table 1.

### 3.2. Regression Analysis

Results of multiple negative binomial regression analysis are shown in Table 2 (second column: with the number of GP visits as outcome measure; third column: with the number of specialist visits as outcome measure). Pseudo R^2^ was 0.05 in the first case and 0.04 in the latter case.

Adjusting for other factors, multiple negative binomial regressions showed that the number of GP visits was positively associated with lower educational level (currently in school training education vs. upper secondary school, IRR: 1.50, 95% CI: 1.01–2.22), being retired (compared to full-time employed, IRR: 1.20, 95% CI: 1.06–1.36), lower levels of loneliness (IRR: 0.93, 95% CI: 0.866–0.998), the presence of at least one chronic condition (IRR: 1.57, 95% CI: 1.44–1.71), lower self-rated health (IRR: 0.75, 95% CI: 0.71–0.79), being vaccinated against COVID-19 (IRR: 1.31, 95% CI: 1.13–1.51), and the presence of depression (IRR: 1.21, 95% CI: 1.05–1.40). Moreover, negative binomial regressions showed that the number of specialist visits was positively associated with being female (compared to being male: IRR: 1.37, 95% CI: 1.20–1.57), being diverse (IRR: 3.12, 95% CI: 1.37–7.09), a lower age (IRR: 0.99, 95% CI: 0.987–0.997), having children (IRR: 1.19, 95% CI: 1.04–1.35), being married (IRR: 1.14, 95% CI: 1.01–1.29), being retired (compared to full-time employed, IRR: 1.58, 95% CI: 1.30–1.90), employment status other than full-time (compared to full-time employed, IRR: 1.21, 95% CI: 1.04–1.41), the presence of at least one chronic condition (IRR: 1.89, 95% CI: 1.65–2.15), lower self-rated health (IRR: 0.75, 95% CI: 0.69–0.82), the presence of depression (IRR: 1.25, 95% CI: 1.04–1.51), being vaccinated against COVID-19 (IRR: 1.34, 95% CI: 1.06–1.69) and lower coronavirus anxiety (IRR: 0.97, 95% CI: 0.95–0.99).

## 4. Discussion

Using data from the general adult population in March 2022, the aim of this study was to investigate the determinants of outpatient physician visits in Germany during later stages of the COVID-19 pandemic. Regressions showed that the number of GP visits was associated with predisposing characteristics and particularly associated with enabling resources and need factors. Moreover, negative binomial regressions showed that the number of specialist visits was associated with all dimensions of the extended Andersen model (i.e., predisposing characteristics, enabling resources, need factors and psychosocial factors).

We identified that the average number of GP visits equaled 2.6 visits and the average specialist visits in the past 12 months equaled 2.1 visits. This is comparable to prior findings. For instance, Hadwiger et al. showed an average number of (total) outpatient physician visits of 4.6 (SD: 5.5) in the general adult population in Germany in the 2000s and early 2010s [16]. After the first stage of the COVID-19 pandemic, this may indicate that the average number of outpatient physician visits is returning to its “natural” level in the German adult population. This may also indicate that the number of individuals postponing their doctor’s visits in Germany during the pandemic (as shown in mid-2020 [3]) is decreasing. However, future research (particularly based on longitudinal data) is required in this area.

Our study demonstrated that while need factors are still key drivers of the number of outpatient physician visits, other factors are also of importance. The association between need factors and HCU is well documented in the literature [17] and can be explained by various illness symptoms that urge individuals to visit the doctor. Various former studies (for example: [18,19]) demonstrated the importance of need factors for physician visits. When outpatient physician visits are solely driven by need factors, this can indicate that outpatient physician services are used appropriately (i.e., if medically necessary).

With regard to predisposing characteristics, several determinants were significantly associated with the outcomes (particularly with the number of specialist visits). For instance, in accordance with former research conducted prior to the pandemic, age was negatively associated with specialist visits [20]. This may be explained by the fact that older individuals commonly regularly visit GPs instead of specialists to meet their health needs [20]. Prior research also demonstrated an association between being female and a higher number of physician visits (e.g., [21]). Furthermore, individuals with children in their own household had a higher number of specialist visits in our study. Given the fact that individuals with children may face various challenges during the pandemic (e.g., fulfilment of multiple roles, e.g., bringing up children while working full time at the same time) [22], we assume this could result in an increased number of specialist visits in this group (e.g., using mental health services) [22]. Moreover, another possible explanation may be that they have a higher number of communicable infectious diseases including COVID-19. Due to the measures to reduce social contacts (home office etc.), contacts took place disproportionately in families and schools. Thus, infections may have mainly occurred in large families with children and in schools. Our findings may thus indicate that particularly individuals with children in their own household are in particular need for assistance. More generally, our study demonstrated the importance of predisposing characteristics for outpatient physician visits—which may be a sign for over- or underuse of such services.

With regard to enabling resources, it appears quite counterintuitive that lower loneliness levels are associated with higher GP visits in our study because we initially assumed that GP visits may substitute missing social contacts (even during the pandemic). However, a recent systematic review also concluded that there is inconclusive evidence regarding the association between loneliness and outpatient physician visits [23]. Possible explanations for the association identified in our study may refer to the fact that individuals with higher loneliness levels may become phlegmatic (in terms of visiting the GP), whereas individuals with lower loneliness levels may be physically active and may have quite frequent preventive visits to the GP [24]. Moreover, individuals already retired reported a higher number of outpatient physician visits compared to full-time employed individuals. This may be particularly explained by the greater amount of leisure time required for such visits. Full-time employed individuals, in contrast, may have multiple obligations to fulfill—particularly during the pandemic. Generally, the relevance of enabling resources for outpatient physician services can indicate inequalities in using outpatient physician services. Upcoming research is required to clarify the role of enabling resources for outpatient physician visits in Germany in further detail.

With regard to psychosocial factors, a higher coronavirus anxiety was associated with a lower number of specialist visits in our study. This is very plausible, as individuals with a high coronavirus anxiety may be afraid of a COVID-19 infection while on their way to the specialist’s office [1]. It is difficult to compare our findings regarding the association between higher coronavirus anxiety and outpatient physician visits with previous studies due to a lack of previous quantitative studies. Future research is required to clarify why coronavirus anxiety was not associated with the number of GP visits. Generally, our study showed the importance of psychosocial factors (in terms of coronavirus anxiety) for specialist visits. Efforts to reduce high levels of coronavirus anxiety may also contribute to specialist visits.

Some strengths and limitations of our study are worth keeping in mind. We clarified the determinants of outpatient physician visits during later stages of the pandemic. A large, representative sample (with regard to sex, age group and state) was used for this study. Common questions were used to quantify outpatient physician visits. We also used a common recall period [25]. The independent variables were selected using the established Andersen model. We also included psychosocial factors. Our study is restricted to individuals up to 74 years. Thus, future research among the oldest individuals is important. Moreover, this is a cross-sectional study with known shortcomings with regard to directionality.

In conclusion, while our study showed that need factors are still a main driver of outpatient physician visits, our findings additionally showed that predisposing characteristics, enabling resources and psychosocial factors are also important for the number of outpatient physician visits in Germany during the COVID-19 pandemic. Knowledge about these determinants (e.g., vaccination status, loneliness or coronavirus anxiety) is also important to avoid under- or overuse. Future research could therefore focus on individuals at risk of underuse or overuse. In particular, treating individuals at risk of overuse can help avoid overburdening the healthcare system, while addressing individuals at risk of underuse may assist in avoiding unmet needs, which in turn may contribute to overall health.

## Figures and Tables

**Table 1:** Sample characteristics.

Variables	Mean (SD)/*n* (%)
**Outcomes**	
GP visits	2.6 (3.6)
Specialist visits	2.1 (3.6)
**Predisposing characteristics**	
Gender	
Male	1554 (50.3%)
Female	1531 (49.5%)
Diverse	6 (0.2%)
Age	46.5 (15.3)
Children in own household	
No	2158 (69.8%)
Yes	933 (30.2%)
Marital status	
Single/Divorced/Widowed/Married, not living together with spouse	1266 (41.0%)
Married, living together with spouse	1825 (59.0%)
Education	
Upper secondary school	1234 (39.9%)
Qualification for applied upper secondary school	356 (11.5%)
Polytechnic Secondary School	196 (6.3%)
Intermediate Secondary School	956 (30.9%)
Lower Secondary School	327 (10.6%)
Currently in school training/education	16 (0.5%)
Without school-leaving qualification	6 (0.2%)
Migration background	
No	2721 (88.0%)
Yes	370 (12.0%)
**Enabling resources**	
Employment status	
Full-time employed	1365 (44.2%)
Retired	646 (20.9%)
Other	1080 (34.9%)
Loneliness (from 1 to 4, with higher values corresponding to higher loneliness)	2.1 (0.7)
**Need factors**	
Chronic diseases	
Absence of at least one chronic disease	1673 (54.1%)
Presence of at least one chronic disease	1418 (45.9%)
Self-rated health (from 1 = very bad to 5 = very good)	3.6 (0.9)
Probable depression	
Absence of probable depression	2377 (76.9%)
Presence of probable depression	714 (23.1%)
Probable anxiety	
Absence of probable anxiety	2595 (84.0%)
Presence of probable anxiety	496 (16.0%)
Being vaccinated against COVID-19	
Not being vaccinated	365 (11.8%)
Being vaccinated	2726 (88.2%)
**Psychosocial factors **	
Coronavirus anxiety (from 0 to 20, with higher values corresponding to higher coronavirus anxiety)	1.4 (3.1)

**Table 2:** Determinants of outpatient physician visits. Results of multiple negative binomial regression analysis.

Independent Variables	Number of GP Visits	Number of Specialist Visits
**Predisposing characteristics:**		
Gender: -Female (Ref.: Male)	1.06	1.37 ***
	(0.97–1.16)	(1.20–1.57)
-Diverse	1.04	3.12 **
	(0.41–2.64)	(1.37–7.09)
Age	1.00	0.99 **
	(1.00–1.00)	(0.99–1.00)
Children in own household: Yes (Ref.: No)	1.07	1.19 *
	(0.96–1.19)	(1.04–1.35)
Marital status: Married, living together with spouse (Ref.: Single/Divorced/Widowed/Married, not living together with spouse)	1.02	1.14 *
	(0.93–1.12)	(1.01–1.29)
Education: -Qualification for applied upper secondary school (Ref.: Upper secondary school)	1.02	0.97
	(0.91–1.14)	(0.82–1.16)
-Polytechnic Secondary School	1.07	0.89
	(0.92–1.25)	(0.72–1.09)
-Intermediate Secondary School	1.02	1.01
	(0.92–1.13)	(0.88–1.16)
-Lower Secondary School	1.10	0.85
	(0.94–1.29)	(0.66–1.08)
-Currently in school training/education	1.50 *	0.52 ^†^
	(1.01–2.22)	(0.24–1.09)
-Without school-leaving qualification	1.10	0.30 ^†^
	(0.58–2.08)	(0.07–1.22)
Migration background: Yes (Ref.: No)	1.00	1.00
	(0.88–1.14)	(0.83–1.20)
**Enabling resources:**		
Employment status: -Retired (Ref.: Full-time employed)	1.20 **	1.58 ***
	(1.06–1.36)	(1.30–1.90)
Other	1.03	1.21 *
	(0.93–1.15)	(1.04–1.41)
Loneliness (from 1 to 4, with higher values corresponding to higher loneliness)	0.93 *	0.97
	(0.87–1.00)	(0.87–1.08)
**Need factors:**		
Chronic diseases: Presence of at least one chronic disease (Ref.: Absence of at least one chronic disease)	1.57 ***	1.89 ***
	(1.44–1.71)	(1.65–2.15)
Self-rated health (from 1 = very bad to 5 = very good)	0.75 ***	0.75 ***
	(0.71–0.79)	(0.69–0.82)
Probable depression: Presence of probable depression (Ref.: Absence of probable depression)	1.21 **	1.25 *
	(1.05–1.40)	(1.04–1.51)
Probable anxiety: Presence of probable anxiety (Ref.: Absence of probable anxiety)	0.99	0.98
	(0.86–1.15)	(0.80–1.20)
Being vaccinated against COVID-19 (Ref.: Not being vaccinated against COVID-19)	1.31 ***	1.34 *
	(1.13–1.51)	(1.06–1.69)
**Psychosocial factors: **		
Coronavirus anxiety (from 0 to 20, with higher values corresponding to higher coronavirus anxiety)	0.99	0.97 **
	(0.98–1.01)	(0.95–0.99)
Constant	4.10 ***	2.89 ***
	(2.80–6.02)	(1.64–5.08)
Observations	3091	3091
Pseudo R^2^	0.05	0.04

Incidence Rate Ratios are reported; 95% CI in parentheses; *** *p* < 0.001, ** *p* < 0.01, * *p* < 0.05, ^†^ *p* < 0.10.

## Data Availability

The datasets used and analyzed during the current study are available from the corresponding author on reasonable request for all interested researchers.
